# Women’s development group leaders’ promotion of maternal, neonatal and child health care in Ethiopia: a cross-sectional study

**DOI:** 10.1080/16549716.2020.1748845

**Published:** 2020-05-27

**Authors:** Fisseha Ashebir, Araya Abrha Medhanyie, Afework Mulugeta, Lars Åke Persson, Della Berhanu

**Affiliations:** a Tigray Regional Health Bureau, Mekelle, Ethiopia; b College of Health Sciences, School of Public Health, Mekelle University, Mekelle, Ethiopia; c London School of Hygiene & Tropical Medicine, London, UK; d Ethiopian Public Health Institute, Addis Ababa, Ethiopia

**Keywords:** Community health volunteers, health extension program, knowledge, performance, training

## Abstract

**Background:**

Women’s development group leaders are volunteer community health workers in Ethiopia who, among other duties, promote health and prevention of diseases. They link and extend essential health services from health posts to households.

**Objective:**

To assess the characteristics, knowledge, and practice of women’s development group leaders in the field of maternal, neonatal, and child health care.

**Method:**

This study used a cluster-sampled cross-sectional survey conducted from December 2016 to February 2017 in four regions of Ethiopia: Oromia, Amhara, Tigray and Southern Nations, Nationalities and Peoples. One of the volunteers, who was available at the time of the survey, was included from each cluster. A total of 187 women’s development group leaders participated in this quantitative study.

**Result:**

Close to half of the women’s development group leaders were illiterate. The leaders had a wide variation in the number of women in their groups. Two-thirds had received some training during the last year, covering a broad range of health topics. Their knowledge of maternal, newborn, and child health was relatively low. Two-thirds had monthly contact with health extension workers. Around half had interacted with other local stakeholders on maternal and child health matters during the last three months. Two-thirds had visited pregnant women, and half had made home visits after delivery in the previous quarter. Activities regarding sick newborns and under-five children were less frequent.

**Conclusion:**

The women leaders were given a wide range of tasks, despite having a low educational level and receiving training through brief orientations. They also showed limited knowledge but had a relatively high level of activities related to maternal health, while less so on neonatal and child health.

## Background

Community health workers (CHWs) provide primary health care and have contributed to health promotion for more than half a century [1,2]. To this effect, they are critical members of the primary health team [3–5]. In Ethiopia, since the introduction of the Health Extension Program and the establishment of the Health Extension Worker (HEW) cadre in 2003, there have been several positive changes in bringing health services closer to the rural population [6,7]. In 2009, only 10% of Ethiopian women gave birth in a health facility. Maternal and neonatal mortality and death rates in children from preventable diseases, like pneumonia, were all high [8,9].

To meet the Millennium Development Goals of reducing maternal and under-five mortality and aligned to the Ethiopian Growth and Transformation Plan, a women-centered community volunteer organization that could support the health system was critical [10,11]. Discontinuing the mixed-gender CHWs model, in 2010, the Ethiopian government introduced a Women’s Development Group (WDG) strategy to provide support and community ownership of primary health care activities, particularly maternal health services. WDG leaders are volunteers, similar to CHWs in other low- and middle-income countries, who volunteer to promote health [6,11]. There are two levels of the Women’s Development Groups. First, 25 to 30 women are organized to form a Women’s Development Group. Second, a group of neighboring six women is further classified into a 1-to-5 network, where one woman is nominated by the members to lead the network [6]. The 1-to-5 network leaders choose a leader among themselves who manages their WDG. The main selection criterion for being a leader is having graduated as a ‘model family’. A model family has fulfilled 12 or more of the 16 health extension program components, which are categorized into family health, disease prevention and control, hygiene and sanitation, and health education and communication. Potential WDG leaders should also be influential members of the immediate community, have experience as a network leader, be trusted by the members, and volunteer to serve as a leader [12]. Some education was a desired but not essential criterion [13]. The WDG strategy has been given different names, and it has been modified over time. Its focus on health matters became broader and included other sectors of development. Further details based on our literature review are summarized in Box 1.

**Box 1.** The Women’s Development Group strategy.

The Ethiopian government introduced the Women’s Development Group (WDG) strategy in Tigray region in 2010, and expanded it to Southern Nations, Nationalities and Peoples, Oromia, and Amhara regions in 2011 [8]. The aim of the WDG strategy was to support the health extension program by encouraging families to take responsibility for their own health and improve community ownership of primary health care. The WDG leaders are also expected to provide support to other sectors, such as agriculture, education, water, justice and good governance activities, women’s affairs and women’s association activities [6]. Their activities include providing food for children, motivating pregnant women to deliver at health institutions, and mobilizing funds to cover transport costs for women in labor [6]. The Women’s Development Groups have also been labeled Women’s Development Army and Health Development Army [10]. The WDG program was originally under the Federal Ministry of Health but from 2016, over site of the group was transferred to the Ministry of Women’s and Children’s Affairs [12].

As close neighbors who socialize regularly, the 1-to-5 leaders are expected to meet with all members of their network every other day. Similarly, the five network leaders of a WDG should meet weekly, and all network leaders should meet with the HEWs every fortnight [12].

The Government of Ethiopia implemented various activities to strengthen the WDG leaders’ knowledge and improve their practices, mainly by developing a WDG manual, organizing annual training [12], distributing job aids, and recognizing good performers [6]. When comparing results from 2010 to 2015, the Federal Ministry of Health has reported that WDG leaders have successfully mobilized women to seek care for maternal, neonatal, and child health services [14]. During this period, facility deliveries increased from 10% to 26% [15]. Recent reports have, however, indicated that the performance of WDG leaders has been unsatisfactory [14]. This problem might have influenced the reported relative low coverage of postnatal care and the low utilization of services provided under the integrated Community Case Management of childhood illnesses and the Community Based Newborn Care [15,16].

Enhancing the performance of the WDG leaders can benefit the social mobilization and participation of the communities, particularly the utilization of maternal, newborn, and child health services. Their performance is affected by the health system, intervention designs, and contextual, as well as individual-level factors [17,18]. The current status and performance of the WDG leaders in Ethiopia are not well documented. Thus, this study aimed to describe the characteristics of the WDG leaders and to assess their knowledge and practice of health promotion of maternal, newborn, and child health services.

## Methods

### Study design

This study was a cluster-sampled cross-sectional survey, which served as a baseline for the evaluation of the Optimizing the Health Extension Program intervention, which aimed to increase care-seeking behaviour for sick under-five children. The data were collected from December 2016 to February 2017.

### Study setting and population

**Figure 1.**
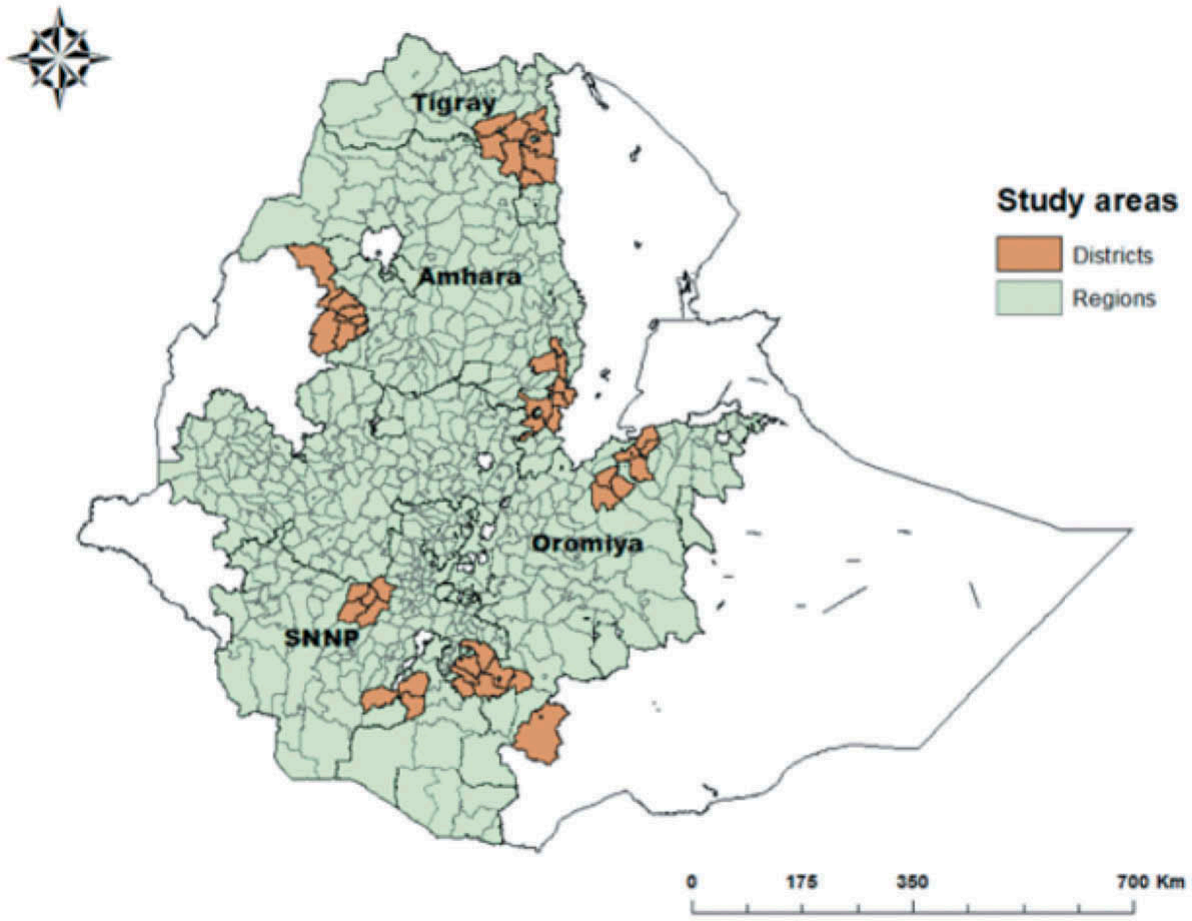
The study woredas in four regions of Ethiopia.

The study areas were Oromia, Amhara, Tigray and Southern Nations, Nationalities and Peoples regions of Ethiopia. From these regions a total of 52 woredas (districts) were included. Figure 1 shows a map of the study woredas. This study targeted the WDG leaders in selected clusters located in the 52 woredas.

### Sampling and data collection

A two-stage stratified cluster sampling method was used. First, the 2007 Ethiopian Housing and Population Census data were used to identify and list enumeration areas in the 52 study woredas. The plan was to select 200 enumeration areas proportional to the size of the woredas. However, six clusters were excluded due to civil unrest, and 194 enumeration areas were finally considered for the study. This study was linked to a household survey. In each enumeration area, 30 households were selected, and one WDG leader,who was available on the day of the study was invited to the interview. If more than one leader was available, the one who had more group members residing in the selected 30 households was prioritized. If unavailable, a WDG leader from the same cluster who had members outside the selected 30 households and who was available on the day of the survey was included. The HEW at the local health post assisted in identifying the WDG leader.

Data collectors were recruited by the Ethiopian Public Health Institute and had, as a minimum, completed their first degree in health sciences. Over the course of two weeks the data collectors were trained on study procedures, the questionnaires, interview and data collection techniques, quality assurance procedures, and study ethics.

A questionnaire was prepared in English and translated into the local languages of Amharic, Oromiffa, and Tigrigna and, after that, back-translated into English. The questionnaire was based on earlier validated survey instruments used at the primary care level in Ethiopia.

The following aspects regarding WDG leaders were captured: 1) Their background characteristics, including age, years of education, years of service, and the number of households they served; 2) Their training on maternal, newborn and child health provided by the HEWs in the past 12 months; 3) Their knowledge on pregnancy-related danger signs, components of essential newborn care, the timing of postnatal care visits in the first six weeks, danger sings in newborns, and general danger sings in under-five children. Knowledge was assessed through unprompted questions and through flashcards depicting images from the family health guide that illustrated key messages to promote maternal, newborn and child health [19]; 4) WDG leaders’ practices were assessed in three ways: 1) Individual promotion of activities, such as promotion of antenatal care for pregnant women, home visits for women who had recently delivered, care-seeking for sick newborns and sick children, 2) Inter-sectoral networking,including questions on their engagement with stakeholders outside the health system to promote maternal and child health; and 3) Community mobilization, i.e. group activities undertaken with HEWs during in three months before the survey to plan events, organize pregnant women’s conferences, provide household visits, conduct health campaigns, and discuss referral of women and children.

The quality of the information collected was ensured by using validated and pretested forms, a system of field supervision, and careful data quality control and management that included daily checks on completeness and consistency. A detailed survey manual with extensive standard operating procedures was prepared and used in training, piloting, and fieldwork.

### Data management and analysis

Data were collected on personal tablet computers, and the Census and Survey Processing System (CSPro) was used to program devices. The collected data were regularly sent to the central server at the Ethiopian Public Health Institute. The server was password-protected, and access to the data was limited to the study team. Data were cleaned and prepared for analysis.

Descriptive statistics, such as frequencies, percentages, and means, were used to analyse the demographic characteristics of the respondents, their knowledge, and practice. We also calculated the proportion of WDG leaders who had undertaken group activities with HEWs and interacted with local stakeholders in the three months preceding the survey. Data were analysed with the Statistical Package for Social Sciences (SPSS) version 20.0 (IBM SPSS Statistics for Windows, Armonk, NY: IBM Corporation).

## Results

### Characteristics of the WDG leaders

A total of 187 (94%) WDG leaders out of the intended 200 WDG leaders were interviewed. Six clusters were excluded due to civil unrest, and in seven clusters, WDG leaders were not available for interviews at the time of the survey. The mean age of the leaders was 36 years, and approximately half of the women had no formal education (Table 1). On average, they had three years of experience as a leader. Despite the program’s model of 1-to-5 and 1-to-30 groupings of households, there was quite a variation in the total number of women the leaders served. Fifty-eight were 1-to-5 leaders, and 89 were 1-to-30 leaders, while the rest were leaders of varying numbers of women.

**Table 1.** Characteristics of Women’s Development Group leaders.

Characteristic	N (%)
**Age**	**N = 186**
≤24 years	21 (11.3)
25–34 years	58 (31.2)
35–44 years	69 (37.1)
≥45 years	38 (20.4)
**Education level**	**N = 187**
No education	92 (49.2)
1 to 4 years	33 (17.6)
5 to 8 years	41 (21.9)
≥9 years	21 (11.2)
**Experience as WDG leader**	**N = 187**
<2 years	72 (38.5)
3 to 4 years	69 (36.9)
≥5 years	46 (24.6)
**Women serve by WDG leader**	**N = 178**
≤10	58 (32.6)
11 to 24	11 (6.2)
25 to 35	89 (50.0)
≥36	20 (11.2)

### Training

Two-thirds (64%) of the WDG leaders had received some training from the HEWs during the last 12 months preceding the survey. The most common topic of training reported by the WDG leaders was the promotion of institutional delivery (60%). Leaders less frequently reported receiving training on topics related to sick newborns or older children with illnesses as compared to maternal health issues (Table 2).

**Table 2.** Training provided by the Health Extension Workers to Women’s Development Group leaders.

Training component	Training provided (N = 119)
	n (%)
Identify and report pregnant women to Health Extension Workers	104 (87.4)
Educate on pregnant women’s danger signs	101 (84.9)
Refer for antenatal care at health facilities	102 (85.7)
Educate pregnant women on birth preparedness	99 (83.2)
Promote facility-based delivery	113 (95.0)
Provide home visits for recently delivered women	96 (80.7)
Refer for postnatal care	92 (77.3)
Identify and educate on newborn danger signs	93 (78.2)
Refer sick newborns	85 (71.4)
Identify and educate on child (2–59 months) danger signs	89 (74.8)
Use of the family health guide	88 (73.9)

### WDG leaders’ knowledge on maternal, newborn, and child health care

**Figure 2.**
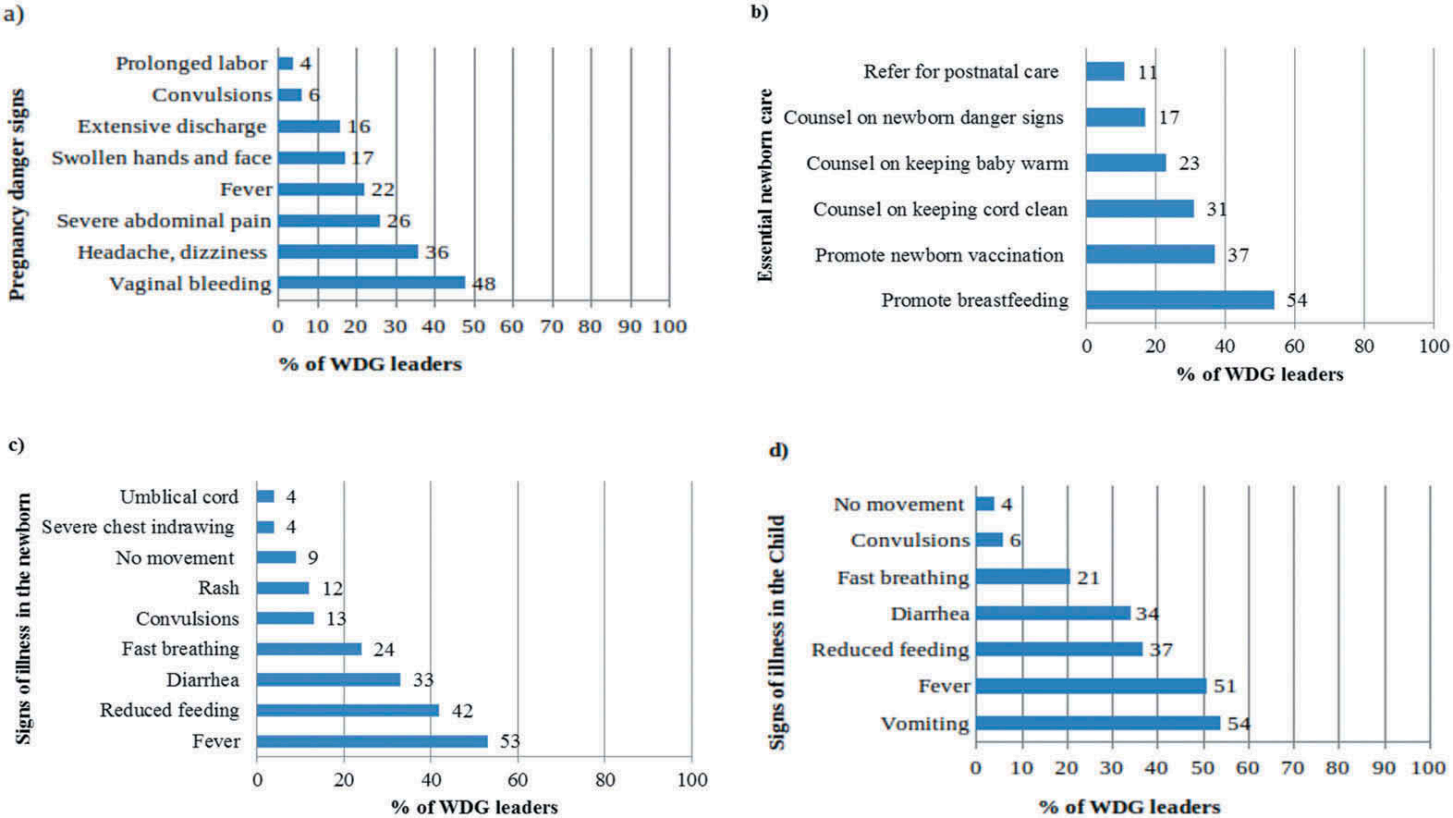
Unprompted knowledge of the Women’s Development Group leaders.

The WDG leaders’ unprompted knowledge of danger signs in pregnant women is displayed in Figure 2(a). Each of the danger signs was mentioned by less than half the study participants, with 48% listing vaginal bleeding and 4% answering prolonged labour.

Few WDG leaders knew the timing of postnatal visits. One quarter mentioned the visits on day one and day three, and one-fifth the 7^th^-day visit. The visit on day 42 was mentioned by 42% of WDG leaders.

The leaders’ unprompted listing of areas of newborn health promotion or counselling was relatively limited; half mentioned the promotion of breastfeeding, but fewer stated vaccinations, cord care, temperature control, newborn danger signs, or referral for postnatal care (Figure 2(b)).

The unprompted knowledge of signs of illness in newborns (Figure 2(c)) or children 2–59 months of age (Figure 2(d)) was also low. Half of the leaders mentioned fever, but other signs and symptoms were infrequently stated.

A family health guide is a tool with pictures and health messages developed for the Ethiopian primary care level. Two-thirds (63%) of the WDG leaders reported having used this tool (Table 3). Among them, the correct identification of the pictorial messages ranged from 17% to 88%. A newborn with a respiratory problem was the least correctly identified message, while most leaders recognized the image of a child being immunized.

**Table 3.** Women’s Development Group leaders’ understanding of key family health guide images of maternal, neonatal, and child health.

Family health card messages	WDG leaders N = 119 (%)
**Pregnancy period**	
Use of iron tablets during pregnancy	46 (38.7)
Use of tablets to treat parasite infestation	26 (21.8)
HIV testing for the couple	45 (37.8)
Pregnancy danger sign: swelling of face and hands (oedema)	65 (54.6)
Pregnancy danger sign: fever	68 (57.1)
Birth preparedness: clean thread, razor, etc.	42 (35.3)
**Delivery period**	
Reporting home delivery to HEW immediately	22 (18.5)
**Newborn and infant care**	
Washing hands with soap before handling newborn	105 (88.2)
Delaying bathing of newborn 24 hours	32 (26.9)
Breastfeeding an infant night time	79 (66.4)
Giving the child vitamin A	85 (71.4)
Vaccinating children	102 (85.7)
Not apply cow dung and others	31 (26.1)
baby’s certificate of vaccination	71 (59.7)
**Newborn and child illness**	
Newborn danger sign: lethargy or unconsciousness	30 (25.2)
Newborn danger sign: fast breathing or grunting	20 (16.8)
Newborn danger sign: umbilical pus, other signs of skin infection	50 (42.0)
Increased breastfeeding of sick babies (0–6 months)	57 (47.9)
Increased breastfeeding and foods to sick babies (≥6 months)	77 (64.7)

### Performance of WDG leaders

#### Promotion of maternal, newborn and child health care

The WDG leaders are expected to educate women on essential maternal, newborn, and child health care and services. In the three months before the survey, 42% of the WDG leaders had adhered to the recommended monthly meetings with the women they served. In the same period, two thirds (61%) had counselled pregnant women to attend antenatal care, and a half (50%) had visited newly delivered mothers at home. Furthermore, 15% of the WDG leaders reported visiting a sick newborn and 20% visiting a sick child. One-third (31%) had reported their maternal, newborn, or child health activities to the HEWs during the three months before the survey, and the same proportion had forms to communicate information from the community to the HEWs.

#### Inter-sectoral networking

Networking with stakeholders outside the health system for maternal, newborn, and child health promotion is one of the expected tasks of WDG leaders. One-third (35%) had interacted with the women’s saving groups, and the same proportion had met with religious leaders. Less than a third (29%) had engaged with an *Edir* (a neighbourhood association), a similar percentage (28%) with the local political administration, and one-fifth (18%) with traditional birth attendants. More than half (55%) had networked with one or more of these stakeholders during the last three months before the survey.

#### Community mobilization

**Figure 3.**
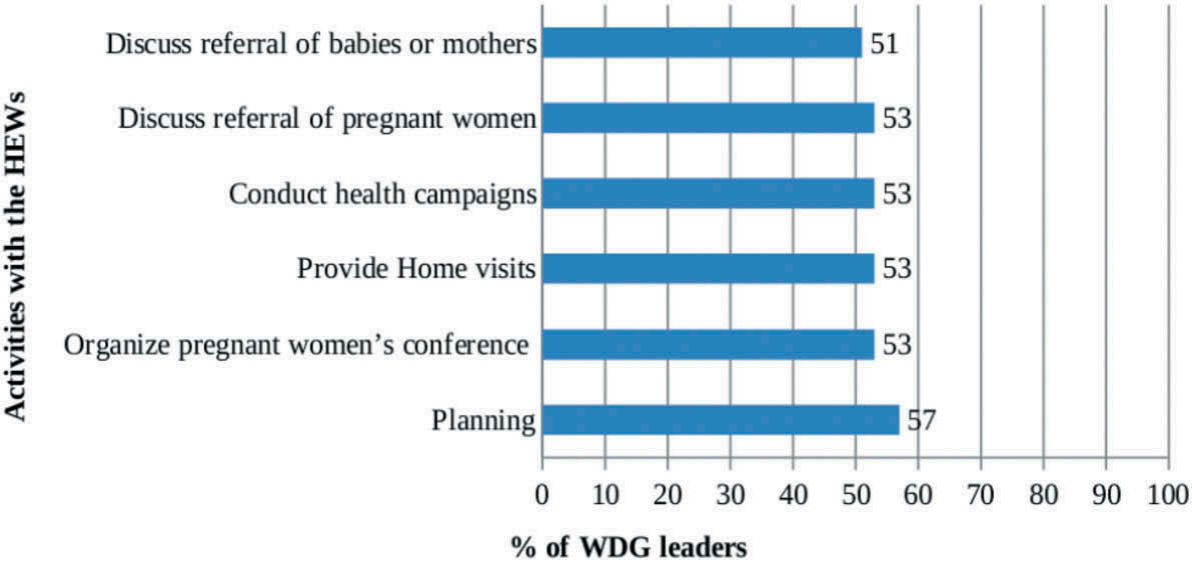
Activities undertaken in the last three months by Women’s Development Group leaders together with Health Extension Workers.

To mobilize the community, WDG leaders facilitate the interaction of women with HEWs. Two-thirds (63%) of the WDG leaders had attended a group meeting with the HEWs in the last quarter before the survey. Figure 3 shows the health-related activities that were undertaken with the HEWs. Less than half (41%) of the WDG leaders had performed all six recommended activities with HEWs during the last three months.

## Discussion

A majority of the WDG leaders had received maternal and child health-related training during the last year, and they demonstrated having some knowledge on women’s health during pregnancy, but less on illnesses in newborns and young children. Most of the leaders had engaged with pregnant women during the quarter before the survey, but their newborn and child health promotion activities were less frequent. Two-thirds of all WDG leaders had interacted with HEWs during the three months before the survey. Half of the WDG leaders had also interacted with other local stakeholders outside the health sector.

In this study, two out of three WDG leaders had received training in the 12 months before the survey, covering a broad range of maternal and child health topics. Training of WDG leaders involves half a day to two days. Given the low educational level of the leaders, a pictorial booklet is used, and the trainings are organized at irregular intervals and for a large number of women each time [6].

The unprompted knowledge of WDG leaders on maternal, newborn, and child health care was relatively low. The leaders mentioned visible danger signs like vaginal bleeding in the pregnant woman or fever in the sick child. Few leaders mentioned rare but vital dangers signs such as prolonged labour in pregnant women, fast breathing, or convulsions in newborns and children. The leaders more frequently recognized maternal and child health danger signs depicted in the family health guide as compared to the answers to the unprompted questions. Some leaders did not have access to the family health guide.

Less than half of the leaders met monthly with women in their groups. Still, most WDG leaders had undertaken health promotion activities for pregnant women and those who had recently delivered. Very few, however, had visited sick newborns or older children, which could partly be due to the few sick newborns or children in their networks.

The establishment of WDG activities in the community has been associated with the increased coverage of maternal health services [20,21] and skilled assistance at delivery [22–24]. There are also indications from studies that WDGs have contributed to higher child immunization coverage [25]. In this study, two-thirds of the WDG leaders had interacted with the HEWs during the month preceding the survey. Half of them had had contact with other groups and stakeholders in the local society to promote maternal, newborn, and child health during the last three months [6].

Over time, the WDG strategy has evolved to have a strong health component with an increasing number of tasks. The oversight of the strategy was transferred to the Federal Ministry of Health and, most recently, to the Ministry of Women’s and Children’s Affairs [12]. At the community level, the organization and management of WDGs have been unclear with variations between geographic areas [20]. These issues could lead to some confusion in the expected roles of these leaders in the community.

### Study strengths and limitations

This relatively large study was conducted in four regions of the country, although without a sample selected to be representative of the regions. The participating WDG leaders had smaller or larger women’s networks. Although not being sampled to represent regions or the entire country, the findings most likely reflect the situation in these four large Ethiopian regions. We have assessed knowledge and activities but did not study the effect on maternal, newborn, or child health outcomes. The recall period was limited to one or three months to reduce the risk of bias.

## Conclusion

This study described the WDG leaders’ training as well as their knowledge and practice in promoting maternal, newborn, and child health. The government of Ethiopia has made a substantial investment in establishing the WDG strategy. The WDG leaders are supporting the HEWs in a range of health activities. Their efforts have, according to several studies, most likely contributed to the improvements in maternal, newborn, and child health outcomes in the country. Despite most WDG leaders being active in their networks, their training was, however, as suboptimal in frequency and content. Further training is needed, especially to improve their newborn and child health knowledge and actions. Given the low educational background of the leaders, innovative educational approaches such as low literacy job aids are required to orient the leaders, which they can also use to promote maternal, newborn and child health in their communities. Modifications may be needed to enhance the WDG strategy for behavioural change in the different cultural contexts across Ethiopia. There might also be a need to revisit the selection criteria for WDG leaders to ensure that they are able to carry out their duties.

## Data Availability

The dataset used in this study can be made available from the Ethiopian Public Health Institute, after contacting Dr. Della Berhanu, email della.berhanu@lshtm.ac.uk.

## Paper context

The women’s development group strategy includes volunteer women who lead groups of women in the local community. This strategy has evolved, and their support to the primary health care for pregnant women, newborns, and children has grown over time. Earlier research showed that active women’s development group may have reinforced the primary health care services. We describe the leaders’ characteristics, knowledge, and health-related activities and suggest some modifications to strengthen this community engagement strategy.
